# Re-evaluation of Laparoscopic Hepatic Subcapsular Spider-Like Telangiectasis Sign: A Highly Accurate Method to Diagnose Biliary Atresia in Infants

**DOI:** 10.3389/fped.2022.850449

**Published:** 2022-04-25

**Authors:** Yibo Li, Liying Rong, Jingfeng Tang, Huizhong Niu, Zhu Jin, Yun Zhou, Guoqing Cao, Xi Zhang, Shuiqing Chi, Shaotao Tang

**Affiliations:** ^1^Department of Pediatric Surgery, Union Hospital, Tongji Medical College, Huazhong University of Science and Technology, Wuhan, China; ^2^Department of Hepatobiliary Surgery, Union Hospital, Tongji Medical College, Huazhong University of Science and Technology, Wuhan, China; ^3^Department of Pediatric General Surgery, Hebei Children’s Hospital of Hebei Medical University, Shijiazhuang, China; ^4^Department of Pediatric Surgery, Affiliated Hospital of Zunyi Medical University, Zunyi, China

**Keywords:** biliary atresia, cholangiography, infant, laparoscopy, diagnosis

## Abstract

**Objective:**

Operative cholangiography, the gold standard for the diagnosis of biliary atresia (BA), is being challenged due to an increase in the studies of misdiagnosis. A previous study has shown that the laparoscopic hepatic subcapsular spider-like telangiectasis (HSST) sign was accurate for diagnosing BA. This study aims to compare the performance of the HSST sign with cholangiography in the identification of BA.

**Methods:**

We prospectively screened consecutive infants with cholestasis who underwent laparoscopic exploration in this multicenter study. Demographics, intraoperative findings (videos and images), and outcomes were retrospectively analyzed. The data of the HSST sign and cholangiography were compared according to the final diagnosis. Then, the diagnostic accuracy of the BA using the HSST sign and cholangiography was validated in other independent cohorts.

**Results:**

A total of 2,216 patients were enrolled in this study. The sensitivity and negative predictive values were both 100% for diagnosing BA based on the HSST sign and cholangiography. The specificity, negative predictive value, and accuracy of the HSST sign (97.2, 99.2, 99.3%) in discriminating BA were significantly higher than operative cholangiography (81.6, 94.9, 95.8; *p* < 0.001). Moreover, to realize the early diagnosis of BA, the accuracy of the HSST sign in identifying BA was better than cholangiography in the subgroup of neonates (98.7% vs. 95.0%; *p* = 0.032). Interestingly, 92 non-BA patients without the HSST sign had positive cholangiography. Among them, 28 infants had negative cholangiography when the common bile duct was compressed and 39 patients displayed visible bile ducts due to repeated postoperative biliary irrigation. The other 25 patients (18 with the Alagille syndrome, 5 with progressive familial intrahepatic cholestasis, and 2 with the neonatal hepatitis syndrome) had consistently positive cholangiography. In the independent validation cohort, the diagnostic accuracy of the HSST sign (99.2%) was higher than cholangiography (95.0%, *p* = 0.012).

**Conclusion:**

The laparoscopic HSST sign is superior to cholangiography in the diagnosis of BA in the infants with cholestasis and has advantages in early diagnosis. This method is expected to become a novel shift for diagnosing BA during ongoing laparoscopy.

## Introduction

Biliary atresia (BA) is characterized by progressive obliteration of the intrahepatic and extrahepatic bile ducts within a few weeks of birth, and it leads to death if not treated before the patient is at the age of 2 years (1, 2). For infants with BA, a timely and accurate diagnosis improves the long-term prognosis of the native liver following Kasai portoenterostomy (KPE) (3). Ultrasonography, hepatobiliary scintigraphy, and magnetic resonance cholangiopancreatography (MRCP) were helpful for the preoperative differentiation of BA from other causes of neonatal cholestasis, and liver histological examination can be especially helpful (4, 5). However, sometimes, the liver puncture biopsy findings are ambiguous due to the change from having bile duct proliferation to a gradual disappearance as the inflammation and fibrosis progress (6). Earlier biopsies that lack the typical histological findings of BA can also add to the confusion.

Even now, histological, or anatomical diagnostic tests that can precisely distinguish BA are still needed. Intraoperative cholangiography (which can be undertaken laparoscopically) was considered a definitive and gold standard for diagnosing BA until the twenty-first century (7–9). This technique, however, from the beginning has been reported to lead to misdiagnoses in 1967 (10), and this situation is now revealed by an increasing number of studies (11–17). Furthermore, the Cholestasis Guideline Committee stated that intraoperative cholangiography could be misleading in up to 20% of the cases (18). Despite the shortcoming, there is still no alternative diagnostic method for BA before KPE. Hence, a reliable complementary diagnostic approach is required.

We previously found that laparoscopic hepatic subcapsular spider-like telangiectasis (HSST) sign, a prominent pathological change on the liver surface, was accurate in the diagnosis of BA (19). Although color Doppler ultrasound can display hepatic subcapsular flow (HSF) sign and can usefully identify BA, the main disadvantage is that it is influenced by the experience of sonographers and the physical shielding of the patient’s body (20, 21). The HSST sign is not easy to see with the naked eye. In contrast, the laparoscopic HSST sign is magnified 4–8 times and is more intuitive, stable, and reliable (19). However, it is unclear whether the diagnostic ability of the HSST sign is higher compared with cholangiography, and the ability to distinguish BA in neonatal cholestasis is also unknown.

Therefore, in this multicenter study, a large sample comparative study was used to further evaluate the accuracy of the two diagnostic methods for differentiating BA in infants with cholestasis. To identify neonatal BA, because early diagnosis correlates with the outcome.

## Materials and Methods

### Patients

**FIGURE 1:**
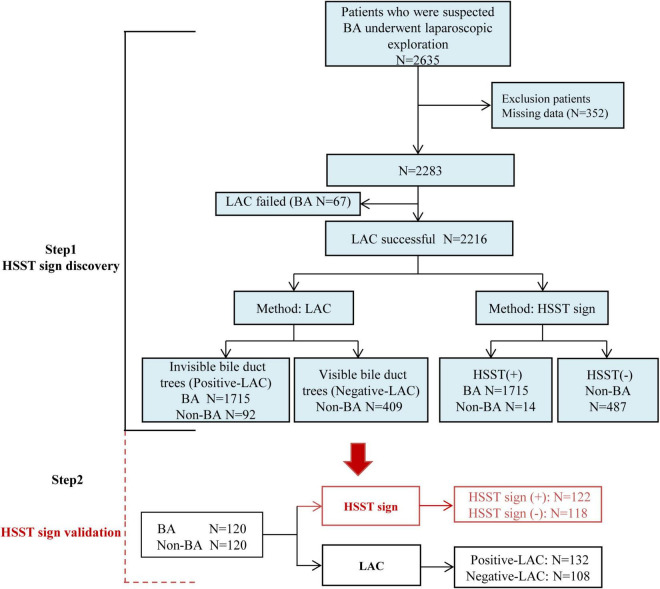
Study design. HSST: hepatic subcapsular spider-like telangiectasis; LAC: laparoscopy-assisted cholangiography; BA: biliary atresia; LAC failed: patients cannot undergo cholangiography due to atrophic gallbladders; Positive LAC: the intrahepatic bile ducts cannot be visualized; Negative LAC: the intrahepatic bile ducts can be visualized.

This study was approved by the Ethics Committee of Union Hospital, Tongji Medical College, Huazhong University of Science and Technology (IORG003571-1). The patients with suspected BA who presented to undergo laparoscopic exploration were prospectively screened in three large tertiary medical centers between January 2005 and December 2018 (Figure 1). The evaluation of cholestasis before surgery included standard liver biochemistry, ultrasound, radiography, and a liver biopsy (when indicated). Laparoscopic exploration for HSST sign and cholangiography was performed in the patients with highly suspected BA. Laparoscopy-assisted cholangiography (LAC) failed in 67 infants and they only underwent laparoscopic exploration due to atrophic gallbladders and different degrees of liver fibrosis. The patients who successfully underwent LAC were established as discovery cohorts. The patients in the negative-LAC group had patent biliary trees and the patients in the positive-LAC group had invisible bile ducts. Informed consent was obtained from parents or guardians before the surgery. All patients were followed up until BA (or non-BA) was confirmed. The criteria for excluding BA met at least one of the following conditions: (1) postoperative pathological examination and cholangiography showing a patent biliary tree, (2) gene detection confirmed etiology, and (3) cholestasis recovered and the stool color normal on follow-up. Demographics, intraoperative findings (videos and images), and outcomes were retrospectively analyzed.

### Hepatic Subcapsular Spider-Like Telangiectasis Sign and Laparoscopic Procedure

**FIGURE 2:**
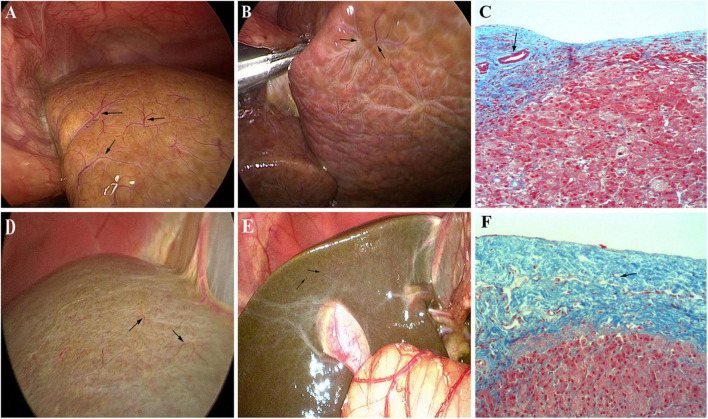
Type of HSST sign: typical HSST sign: **(A)** concentrated type (arrow) of the HSST sign in a 58-day-old male with BA; **(B)** dispersed type (arrow) of the HSST sign in a 61-day-old male with BA; **(C)** dilated arteries (arrow) in the hepatic subcapsular area in BA (Masson; ×100). Atypical HSST sign: Tiny vessel plexuses in a 55-day-old male with AGS **(D)** and in a 51-day-old male with NHS **(E)**. **(F)** Vessels in AGS were revealed as dilated capillaries (arrow) in the hepatic subcapsular region (Masson; ×100).

**FIGURE 3:**
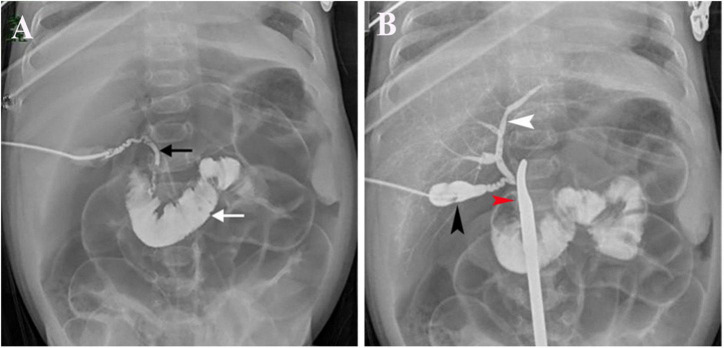
LAC in a 51-day-old patient with cholelithiasis. **(A)** Contrast agents entered the duodenum (white arrow) and showed a discontinuous common bile duct (black arrow). **(B)** Contrast agents were injected from the gallbladder (black arrow) and had visible intrahepatic bile ducts (white arrow) when the inferior segment of the common bile duct was controlled with a forceps (red arrow).

The typical HSST sign was defined as enlarged tortuous spider-like vascular plexuses with four to eight branches that were distributed all over the liver surface under laparoscopy, and these showed either a centralized vascular plexus or scattered type (Figure 2). These vascular clusters were arterial dilation on pathological examination. The atypical HSST sign was presented as (1) tiny blood vessels on the surface of the liver (Figure 2D), (2) no spider-like branches (Figure 2E), and (3) the dilation of capillaries in the hepatic subcapsular region rather than the hepatic artery branches, and these were confirmed by pathology (Figure 2F).

Laparoscopic procedures were performed under general anesthesia. A 5-mm umbilical camera port and a working port with 3-mm forceps (also used for liver biopsy) above the umbilicus were placed separately to assess the gallbladder, to determine if there was liver cirrhosis, and to examine the extrahepatic bile ducts (22). If the extrahepatic bile ducts existed and the gallbladder appeared normal, the bile duct was compressed by gradually and repeatedly inputting saline until no resistance was present. Then, radiocontrast was injected through the gallbladder, and pictures were taken. When the contrast agents entered the duodenum without retrograde filling of the proximal hepatic bile ducts, the inferior part of the common bile duct was temporarily compressed, and the above procedure was repeated. Once the gallbladder and extrahepatic bile duct were atretic resulting in an impossible cholangiogram, a KPE was necessary. In addition, the patients had postoperative liver histopathologic examinations. The HSST sign was carefully examined under a magnified laparoscopic image. If the contrast medium flowed into the duodenum and intrahepatic bile ducts, hepatoportoenterostomy was not performed. If the intrahepatic bile duct was not shown by cholangiography in the patients without the HSST sign, genetic testing could be used to confirmed.

### Validation Protocol

To test the reproducibility of the HSST sign, we performed the assay in other independent cohorts. We enrolled a series of 120 BA and age-matched 120 non-BA patients in the Union Hospital, Tongji Medical College, Huazhong University of Science and Technology from January 2019 and December 2020. The HSST sign was used to identify BA. Then the LAC was recorded and compared. All enrolled patients were diagnosed according to postoperative liver pathology and follow-up.

### Statistical Analysis

The data were analyzed by SPSS software version 22.0. The Student’s *t*-test or ANOVA for continuous data and chi-square test or Fisher’s exact test for categorical data was applied to assess the differences between groups, respectively. A receiver operating characteristic (ROC) curve analysis, that reported the area under the curve (AUC) with a 95% confidence interval (CI), was performed to illustrate the predictive performance. All data were presented as mean ± SD and percentage, a *p*-value < 0.05 was considered statistically significant.

## Results

### Characteristics of the Patients

**FIGURE 4:**
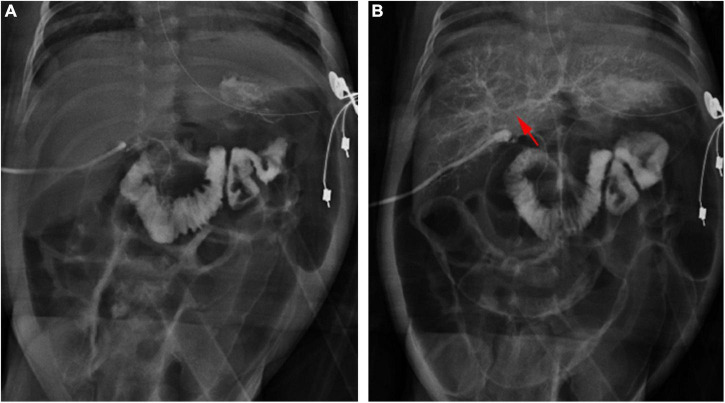
Cholangiography in a 28-day-old patient with TPN cholestasis. **(A)** Positive cholangiography. **(B)** Intrahepatic bile ducts (arrow) were shown after repeated biliary irrigation 1 week postoperatively.

All 1,782 BA patients (including 67 patients who could not undergo cholangiography) had the HSST sign, of which 1,715 had positive LAC (Table 1). A total of nineteen patients were diagnosed with syndromic BA. There were 189 BA patients with cytomegalovirus (CMV) infection (CMV-IgM positive in serum), 1,479 patients with isolated BA, and 28 patients with cystic BA. The typical HSST sign could be observed in all these types. The HSST sign was observed during the operation. The HSST sign could be found in the patients with BA from the age of 18 days to 186 days and was not associated with the age of BA. Although the diameter of the vessels was larger in older BA patients, there were still older children with small vessel diameters. Among 501 non-BA patients in the discovery cohort, 14 cases [9 patients with the Alagille syndrome (AGS) and 5 patients with the neonatal hepatitis syndrome (NHS)] had atypical HSST sign, but they had visible bile ducts using cholangiography. Notably, 92 infants without the HSST sign had positive LAC, and BA was ruled out in these patients. Specifically, 44 patients were diagnosed with AGS, 20 patients were diagnosed with progressive familial intrahepatic cholestasis (PFIC), 15 patients were diagnosed with NHS, 3 patients were diagnosed with total parenteral nutrition (TPN) cholestasis, 3 patients were diagnosed with cholelithiasis, one patient was diagnosed with citrin deficiency, and 6 patients had cholestasis of unknown etiology. Among them, the results of the second LAC (the inferior segment of the common bile duct was controlled) showed that 28 patients had visible intrahepatic bile ducts (Figure 3). For 64 non-BA patients who were still unable to display bile duct trees, an indwelling catheter was performed in the bile duct during the operation. And the bile duct was rinsed with normal saline every day for 1 week after the operation. Then, cholangiography was performed again. The diagnosis was confirmed based on genetic testing and postoperative pathological examination. Of them, 39 infants (AGS in 22 patients, PFIC in 6 patients, TPN cholestasis in 3 patients, NHS in 2 patients, and cholestasis of unknown etiology in 6 patients) displayed visible bile ducts under cholangiography (Figure 4). The other 25 patients (AGS in 18 patients, PFIC in 5 patients, and NHS in 2 patients) had consistently invisible bile duct trees. Patients with AGS or PFIC waited for liver transplantation depending on their parents’ choice. Drug treatment was given for other patients, including ursodeoxycholic acid. They recovered and the stool color was normal on follow-up. Namely, 92 patients were misdiagnosed as false positives (non-BA who were initially misdiagnosed with BA) by LAC but were correctly diagnosed by the HSST sign.

**TABLE 1:** Clinical characteristics and demographics of the patients in the discovery cohort who underwent cholangiography.

Variables	Final diagnosis		*P*-value
	
	BA (*n* = 1715)	non-BA (*n* = 501)	
Age (d)	59 ± 29 (18–186)	57 ± 27 (19–173)	0.591
Sex (female/male)	910/805	241/260	0.051
Weight (Kg)	3.3 ± 0.9	3.6 ± 1.2	0.461
LAC (negative)	0	409	–
LAC (positive)	1715	92	–
Disease type (n)			
Alagille syndrome	–	44	–
PFIC	–	20	–
NHS	–	15	–
Cholelithiasis	–	3	–
Citrin deficiency	–	1	–
TPN cholestasis	–	3	–
Cholestasis of unknown etiology	–	6	–
HSST sign (+)	1715	14	–
HSST sign (–)	0	487	–

*BA: biliary atresia; LAC: laparoscopy-assisted cholangiography; PFIC: progressive familial intrahepatic cholestasis; NHS: neonatal hepatitis syndrome; TPN: total parenteral nutrition.*

### Diagnostic Value of the Hepatic Subcapsular Spider-Like Telangiectasis Sign and Cholangiography for Biliary Atresia

**FIGURE 5:**
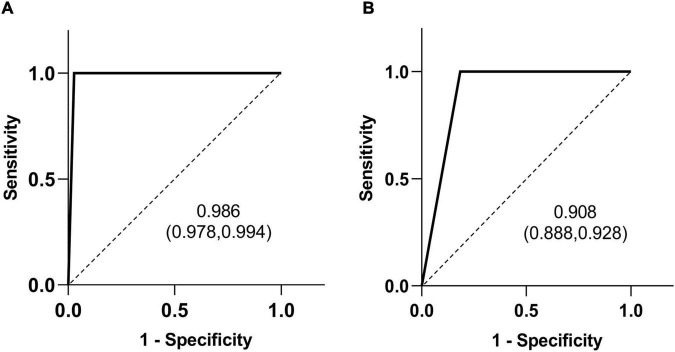
Receiver operating characteristic (ROC) curves of the HSST sign **(A)** and LAC **(B)** in differentiating BA. The area under the curve (AUC) value is shown with the 95% confidence interval (CI).

A typical HSST sign was observed in all the patients who were eventually diagnosed with BA. Therefore, regarding the diagnostic ability of the HSST sign and LAC (Tables 1, 2), both the sensitivity and negative predictive values (NPV) were 100%. The HSST sign displayed significantly higher specificity (97.2% vs. 81.6%, *p* < 0.001), positive predictive value (PPV) (99.2% vs. 94.9%%, *p* < 0.001), and accuracy (99.3% vs. 95.8%%, *p* < 0.001) in discriminating BA than LAC. The AUC for the HSST sign in BA was 0.986 (95% CI: 0.978–0.994; Figure 5A), whereas the AUC for LAC was 0.908 (95% CI: 0.888–0.928; Figure 5B). The neonates at the onset of jaundice and serum liver function values (except γ-GGT) before the exploratory operation showed no statistically significant difference in the non-BA versus BA groups (Table 3). Among the non-BA neonates, 12 had positive LAC but did not have an HSST sign, and 3 had negative LAC but had an atypical HSST sign. Therefore, as shown in Table 4, the specificity, PPV, and accuracy of the HSST sign were better than those of the LAC (96.2% vs. 84.8%, 98.2% vs. 93.1%, 98.7% vs. 95.0%; *p* < 0.05).

**TABLE 2:** Test evaluation between two methods.

Methods	LAC	HSST sign	*P-value*
Sensitivity	100%	100%	–
Specificity	81.6%	97.2%	< 0.001
NPV	100%	100%	–
PPV	94.9%	99.2%	< 0.001
Accuracy	95.8%	99.3%	< 0.001

*LAC: laparoscopy-assisted cholangiography; NPV: negative predictive value; PPV: positive predictive value.*

**TABLE 3:** Clinical data of neonates with BA and non-BA.

Variables	BA group (*n* = 163)	non-BA group (*n* = 79)	*P-value*
Age (d)	26 ± 4 (18–28)	25 ± 4 (19–28)	0.836
Sex (female/male)	88/75	38/41	0.390
Weight (Kg)	2.7 ± 0.6	2.8 ± 0.7	0.378
Direct bilirubin (μmol/L)	201.8 ± 95.3	139.8 ± 71.7	0.055
ALT (U/L)	150.1 ± 53.8	126.2 ± 61.6	0.375
AST (U/L)	231.7 ± 83.8	202.7 ± 71.3	0.292
γ- GGT (U/L)	471.6 ± 122.9	313.6 ± 101.8	0.033*
LAC (negative)	0	67	–
LAC (positive)	163	12	–
HSST sign (+)	163	3	–
HSST sign (–)	0	76	–

LAC: laparoscopy-assisted cholangiography; * p < 0.05.

**TABLE 4:** Test evaluation in neonates.

Methods	LAC	HSST sign	*P-value*
Specificity	84.8%	96.2%	0.027
PPV	93.1%	98.2%	0.032
Accuracy	95.0%	98.7%	0.032

*LAC: laparoscopy-assisted cholangiography; PPV: positive predictive value.*

### Validation of the Hepatic Subcapsular Spider-Like Telangiectasis Sign as a Diagnostic Role for Biliary Atresia

In the validation cohort, a typical HSST sign was observed in all 120 BA patients; 118 non-BA patients had no HSST sign and two patients (cytomegalovirus hepatitis) had atypical HSST sign. All BA patients had positive LAC; 108 non-BA patients had negative LAC. Twelve non-BA patients (6 with AGS, 4 with NHS, 2 with alpha 1 antitrypsin deficiency) had positive LAC and were misdiagnosed with BA. Therefore, the diagnostic accuracy of the HSST sign (99.2%) was higher than cholangiography (95.0%, *p* = 0.012).

## Discussion

The HSST sign was not isolated in the BA patients, but spread all over the surface of the liver, including the diaphragmatic surface and the visceral surface. The distribution characteristics of the HSST sign have no relationship with age. We initially thought the HSST sign was secondary to liver fibrosis of BA (21, 23). However, the HSST sign was also observed in the BA patients aged 18 days, but no liver fibrosis. In our unpublished animal experiments, the HSST sign was observed on the liver surface of the BA mice for 7 days (the rhesus rotavirus-induced BA model has the short lifespan and does not allow obvious liver fibrosis) under microscopy. During the pathological changes of BA, the HSST sign was independent of the age of BA, as it can be observed from the age of 18 to 186 days. Furthermore, we found that the notch3 pathway, predominantly expressed in the vascular smooth muscle cells (VSMCs), was significantly overactivated in both human and experimental BA in the hepatic arterial system. The HSST sign failed to develop in the liver surface of *notch3*^–/–^ BA mice (data unpublished). Therefore, we considered that the virus activated the notch3 pathway and regulated VSMCs, leading to vascular remodeling and abnormal resistance, and this resulted in small artery dilation (24). Thus, the HSST sign occurred because of hyperplastic and hypertrophic changes in the branches of the hepatic artery (23). The microscopic examination enabled us to confirm the presence of dilated vessels, which were hypertrophic hepatic small arteries, in the hepatic subcapsular area (19, 23). Ultrasonic HSF was reflected as an HSST sign by laparoscopy. Lee et al. described that HSF was 100% sensitive and 86% specific to distinguish BA from non-BA disease (20). El-Guindi et al. (21) found that the sensitivity and specificity of HSF were both 96.3%. A meta-analysis showed that HSF could provide high accuracy in predicting BA individuals (23). The HSST sign was previously found to be valuable for diagnosing BA (19). In this multicenter study, we re-evaluated the HSST sign for the diagnosis of BA based on amplified laparoscopic images of a large sample size. The specificity, PPV, and accuracy of the HSST sign were significantly higher in discriminating BA compared with the LAC (97.2% vs. 81.6%, 99.2% vs. 94.9%, 99.3% vs. 95.8%; *p* < 0.001). Moreover, the diagnostic accuracy of the HSST sign (99.3%) was higher than cholangiography (95.0%, *p* = 0.012) in the independent cohort.

It is generally accepted that the earlier the hepatoportoenterostomy is performed, the better the clinical outcomes. Therefore, for infants with conjugated hyperbilirubinemia, an early and accurate diagnosis of BA is mandatory (25). Our results indicated that the HSST sign had excellent performance in the discrimination of BA in neonates with cholestasis, and its diagnostic ability was better than that of LAC (98.7% vs. 95.0%, *p* = 0.032).

Intraoperative cholangiography has been regarded as the gold standard and a definitive investigative test in the diagnosis of BA (26–29). However, different studies have established that cholangiograms are fallible in distinguishing between the BA and non-BA patients. This led to the misdiagnosis of non-BA patients as BA patients, which may be an important reason for the inconsistent postoperative outcomes of Kasai that were reported by different centers. Hays et al. reported that 8% of the patients with neonatal hepatitis had unnecessary KPE due to the erroneous diagnosis of BA by operative cholangiography (10). For differentiating BA from AGS, Emerick et al. (11) reported that only 26% (5/19) of the AGS patients had visible intrahepatic bile ducts on cholangiograms, and this highlights the limitations of intraoperative cholangiography. Han et al. (13) reported that the patent biliary tree of 3 infants with AGS was not presented by intraoperative cholangiography, resulting in a misdiagnosis of BA. Furthermore, 26 patients with AGS from six studies were misdiagnosed with BA and received KPE based on intraoperative cholangiography (14–16, 30–32). Unfortunately, AGS exhibited considerable overlapping features with BA, leading to improper KPE and even worsening the patient’s outcome (33). The misdiagnosis caused by cholangiography in these diseases may be related to the presence of hypoplastic extrahepatic bile ducts and bile plugs (32, 34). Similarly, in our study, the initial cholangiography revealed that 92 non-BA patients had invisible intrahepatic bile ducts. However, when the lower part of the common bile duct was clamped, the cholangiography showed a visible bile duct tree in most non-BA patients. Sixty-four (2.9%) non-BA patients were still unable to display bile duct trees. A few cases had hypoplasia of the extrahepatic bile duct, so the contrast medium could not pass through.

The atypical HSST sign was recorded in 14 non-BA cases (9 with AGS, 5 with NHS) in the negative-LAC group. This feature was validated as dilated capillaries rather than dilated arterioles according to pathologic examination. Furthermore, to verify the sensitivity of the HSST sign, patients who did not undergo cholangiography due to cystic atresia were also reviewed. The HSST sign was discovered in all BA patients under laparoscopy, with a sensitivity of 100%. Typical HSST sign was absent in 5.1% (92/1807) of the patients whose LAC images failed to reveal intrahepatic bile ducts, and BA was eventually excluded in these patients. Deleterious interventions could be avoided in these infants. High-serum metalloproteinase-7 level was a predictive biomarker of BA among infants with cholestasis (35, 36). The metalloproteinase-7 levels in the patients with TPN cholestasis (18-35 ng/mL, 25.6 ± 7.8 ng/mL) overlapped with the BA patients, and could not be used to exclude BA (Supplementary Table 1). Surprisingly, the HSST sign was not detected in these patients, thus ruling out BA. Compounding the dilemma was the fact that 3 of them had positive LAC. Nevertheless, the lack of an HSST sign could still rule out BA. Owing to this good characteristic, using the HSST sign led to only 0.6% (*n* = 14) false-positive results for BA, and this prevented 5.1% (*n* = 92) needless operations, which was superior to cholangiography.

The HSST sign is associated with the following advantages. First, laparoscopy is widespread in almost all hospitals, which makes a diagnosis through simple laparoscopic observation available, and this reduces the degree of the diagnostic difficulty. Images are acquired with a RGB camera and spectral separation is amplified by adapted color processing algorithms, enhancing the interface between vascularized and non-vascularized tissues through Spectra A and Spectra B of Storz Professional Image Enhancement System (Image1 S, Karl Storz, Tuttlingen, Germany). Especially, the HSST sign is highlighted with Spectra B mode (Supplementary Figure 1), as it is based on a color tone shift algorithm to reduce the prevailing red spectral refection, while still preserving the entire spectral information contained in the original color bands. Second, compared with LAC, laparoscopic HSST significantly reduces the diagnosis time during surgery. Third, laparoscopic HSST is less invasive and possibly averts the biliary duct injury and ionizing radiation caused by cholangiography (37, 38).

In conclusion, the present study further investigates the power of the HSST sign for diagnosing BA by comparing it with cholangiography. The absence of intrahepatic bile ducts by cholangiography was not always a “must” for defining BA. The laparoscopic HSST sign is a minimally invasive, radiation-free, and time-saving method to accurately differentiate BA. Additionally, the HSST sign, given its simplicity, stability, and intuitive amplification under laparoscopy, is particularly suitable for primary hospitals with a minimal number of specialists. A future prospective randomized study is needed to identify the diagnostic ability of the HSST sign.

## Data Availability Statement

The original contributions presented in the study are included in the article/Supplementary Material, further inquiries can be directed to the corresponding authors.

## Ethics Statement

The studies involving human participants were reviewed and approved by Ethics Committee of Union Hospital, Tongji Medical College, Huazhong University of Science and Technology. Written informed consent to participate in this study was provided by the participants’ legal guardian/next of kin. Written informed consent was obtained from the minor(s)’ legal guardian/next of kin for the publication of any potentially identifiable images or data included in this article.

## Author Contributions

ST and SC designed the study, reviewed, and revised the manuscript. YL, LR, and JT performed the search, analysis, and wrote the manuscript. HN, ZJ, and YZ collected the data. GC and XZ rechecked the data. All authors contributed to the article and approved the submitted version.

## Conflict of Interest

The authors declare that the research was conducted in the absence of any commercial or financial relationships that could be construed as a potential conflict of interest.

## Publisher’s Note

All claims expressed in this article are solely those of the authors and do not necessarily represent those of their affiliated organizations, or those of the publisher, the editors and the reviewers. Any product that may be evaluated in this article, or claim that may be made by its manufacturer, is not guaranteed or endorsed by the publisher.
