# Supplementary material for: Re-evaluation of Laparoscopic Hepatic Subcapsular Spider-Like Telangiectasis Sign: A Highly Accurate Method to Diagnose Biliary Atresia in Infants
Source: Front Pediatr. 2022 Apr 25;10:850449. doi: 10.3389/fped.2022.850449 (PMC9081763; doi:10.3389/fped.2022.850449)
Supplement: Supplementary file 1 [file Data_Sheet_1.PDF]

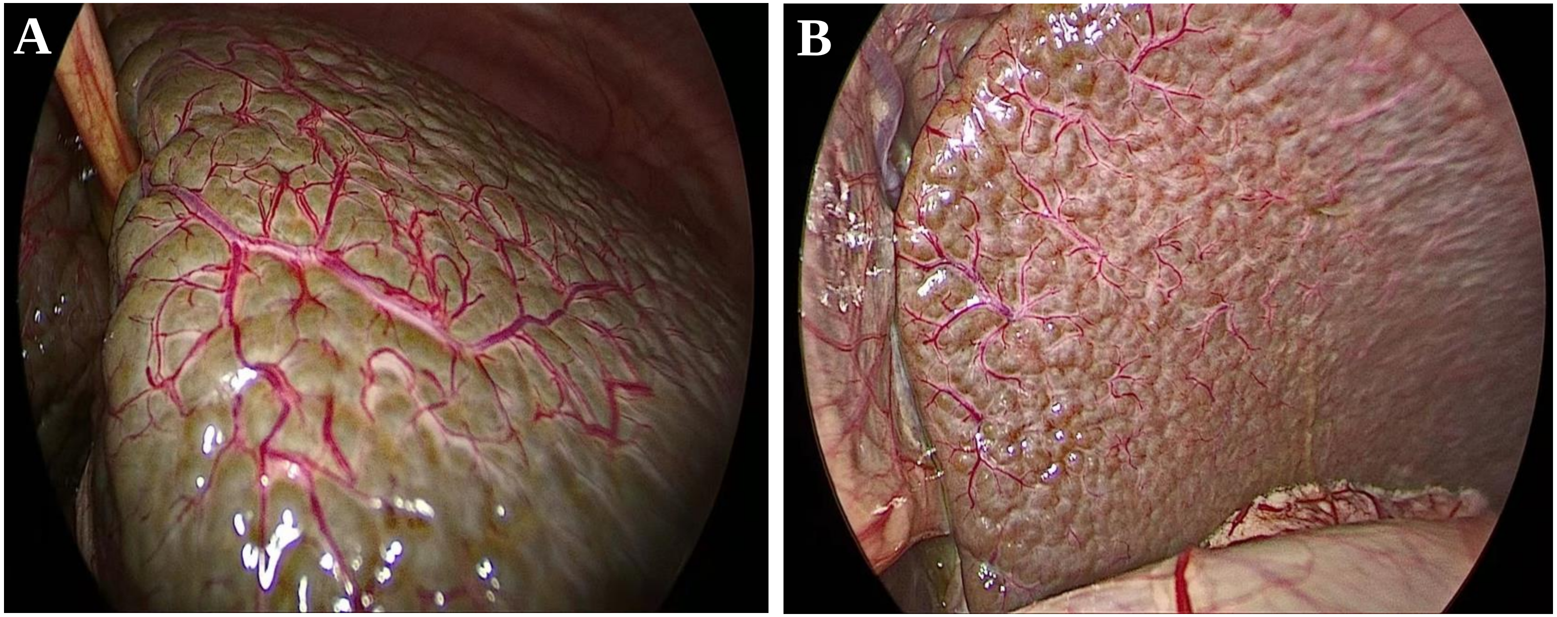

Supplementary Figure 1. HSST sign is highlighted with Spectra B mode. (A: a 62-day-old male with BA; B: a 24-day-old female with BA)
