# Supplementary material for: Re-evaluation of Laparoscopic Hepatic Subcapsular Spider-Like Telangiectasis Sign: A Highly Accurate Method to Diagnose Biliary Atresia in Infants
Source: Front Pediatr. 2022 Apr 25;10:850449. doi: 10.3389/fped.2022.850449 (PMC9081763; doi:10.3389/fped.2022.850449)
Supplement: Supplementary file 2 [file Table_1.DOCX]

**Supplementary Table 1 Clinical data of patients with TPN cholestasis and BA.**

| Variables | TPN cholestasis (n = 38) | BA (n = 52) | *p* |
| --- | --- | --- | --- |
| Age (d) | 53 ± 10 (28-92) | 56 ± 12 (27-95) | 0.379 |
| Sex (female/male) | 16/22 | 28/24 | 0.271 |
| Direct bilirubin (μmol/L) | 132.3 ± 30.9 | 150.8 ± 40.2 | 0.067 |
| γ- GGT (U/L) | 306.2 ± 79.5 | 356.2 ± 101.5 | 0.071 |
| MMP-7 (ng/mL) | 25.6 ± 7.8 (19-35) | 33.8 ± 16.6 (21-123) | 0.041 |
| LAC- positive | 3 | 52 | - |
| HSST sign (+) | 0 | 52 | - |

TPN: total parenteral nutrition; MMP-7: Metalloproteinase-7
